# Increased VEGF-A in solid type of lung adenocarcinoma reduces the patients’ survival

**DOI:** 10.1038/s41598-020-79907-6

**Published:** 2021-01-14

**Authors:** Woon Yong Jung, Kyueng-Whan Min, Young Ha Oh

**Affiliations:** Department of Pathology, Hanyang University Guri Hospital, Hanyang University College of Medicine, Guri, Gyeonggi-do 11923 Republic of Korea

**Keywords:** Cancer, Computational biology and bioinformatics, Drug discovery, Systems biology, Biomarkers, Medical research, Oncology, Pathogenesis

## Abstract

The histological classification of lung adenocarcinoma includes 5 types: lepidic, acinar, papillary, micropapillary and solid. The complex gene interactions and anticancer immune response of these types are not well known. The aim of this study was to reveal the survival rates, genetic alterations and immune activities of the five histological types and provide treatment strategies. This study reviewed the histological findings of 517 patients with lung adenocarcinoma from The Cancer Genome Atlas (TCGA) database and classified them into five types. We performed gene set enrichment analysis (GSEA) and survival analysis according to the different types. We found six oncogenic gene sets that were higher in lung adenocarcinoma than in normal tissues. In the survival analysis of each type, the acinar type had a favorable prognosis, and the solid subtype had an unfavorable prognosis; however, the survival differences between the other types were not significant. Our study focused on the solid type, which had the poorest prognosis. The solid type was related to adaptive immune resistance associated with elevated CD8 T cells and high CD274 (encoding PD-L1) expression. In the pathway analyses, the solid type was significantly related to high vascular endothelial growth factor (VEGF)-A expression, reflecting tumor angiogenesis. Non-necrosis/low immune response affected by high VEGF-A was associated with worse prognosis. The solid type associated with high VEGF-A expression may contribute to the development of therapeutic strategies for lung adenocarcinoma.

## Introduction

Lung cancer is the most frequently diagnosed major cancer and the most common cause of cancer mortality worldwide^1,2^. Among the histological features of lung cancer, adenocarcinoma is the most common type in never-smokers. According to the National Comprehensive Cancer Network (NCCN) clinical practice guidelines in oncology, early lung adenocarcinoma requires surgical resection, but advanced adenocarcinoma is treated with systemic therapy, which is a complete cure option^3^. However, nearly 50% of patients will experience relapse, usually within the first year after initial treatment^4,5^.

Molecular/genetic studies utilize bioinformatics analyses to identify the mechanisms and biomarkers of chemotherapy resistance in order to prevent lung cancer recurrence. In clinical practice, the mutation/expression of epidermal growth factor receptor (EGFR) and anaplastic lymphoma kinase (ALK) in first-line targeted therapy improves the survival rate of patients with lung adenocarcinoma. Programmed death-ligand 1 (PD-L1; encoded by the CD274 gene), a targeted protein in immunotherapy for malignant melanoma, could be an immunotherapy target in lung adenocarcinoma^6,7^.

Along with molecular biological analysis, the 2015 World Health Organization (WHO) classification of the histopathological features of lung adenocarcinoma includes the following five types: lepidic, acinar, papillary, micropapillary and solid^8^. There are several differences in this classification compared with those previously published by the WHO^9–12^. The classification into five types emphasizes the correlations between the pathologic aspect of tumors and the clinical, radiologic, and molecular characteristics^13^. Moreover, this classification stemmed from a multidisciplinary association with pathologists as well as clinicians, radiologists, molecular biologists and surgeons. The histological findings of lung adenocarcinoma are heterogeneous, and various components are mixed^13^. A previous study demonstrated that more than 80% of lung adenocarcinoma was present as the mixed type^14^, but the major type of adenocarcinoma is classified according to the predominant histologic subtype.

Several studies have reported the survival rates of the histological types of lung adenocarcinoma according to the WHO classification. Previous studies demonstrated that a predominant micropapillary type was predictive of poor prognosis in lung adenocarcinoma^15,16^. Another study suggested that a solid predominant type correlated with poor progression-free survival^17^. Another study reported that the histological group including the micropapillary and/or solid type is an independent predictor of early recurrence^18^.

In recent years, next-generation sequencing (NGS) and big data analytics have allowed the analysis of biomarker genes, the quantification of the different types of tumor-infiltrating immune cells and the molecular network-based integration of multiomics data^19–21^. Considering the complex gene-environment interactions of lung adenocarcinoma, including the different histological types, the clinical application of gene expression data is difficult. For these reasons, we believe that analyses using gene expression data should focus on identifying a simple, robust, and druggable marker based on bioinformatics and high-throughput experimental methods to achieve accessible and effective therapeutic strategies. The Cancer Genome Atlas (TCGA) database has a large amount of data, including virtual histological slides and genomic data, such as RNA sequencing, mutation, copy number alteration and methylation data^19^. Moreover, the TCGA database provides data on the five histological types of lung adenocarcinoma classified by pathologists as well as clinical and genetic information.

This study aimed to determine whether the histological types based on the five features contribute to the survival rate of lung adenocarcinoma and to analyze their prognostic value in the TCGA database^22^. We further aimed to identify the gene sets related to the histological types using gene set enrichment analysis (GSEA)^20^ and pathway-based network analysis^23,24^. The distributions of immune cells were analyzed according to the histological types^21^. Using the Genomics of Drug Sensitivity in Cancer (GDSC) and Catalog of Somatic Mutations in Cancer (COSMIC) databases, we performed drug screening in lung cancer cell lines according to the genetic alterations associated with the histological types^25,26^.

## Materials and methods

### Patient selection, gene set enrichment analysis, and pathway-based network analysis based on the TCGA database

We obtained a total of 1053 non-small cell lung carcinoma (NSCLC) cases comprising 566 lung adenocarcinomas and 487 squamous cell carcinomas with known mRNA expression and mutations from the TCGA database^19^. The analysis was performed on 517 cases containing both virtual histopathological slides and genetic data from 566 lung adenocarcinomas.

For detection of significant gene sets, GSEA (version 4.03) was performed with 25,724 gene sets in the Molecular Signatures Database (MSigDB version 7.1) from the Broad Institute at MIT^20^. Specific gene sets (189 oncogenic gene sets) were used to identify those associated with the histological types such as lepidic, acinar, papillary, micropapillary and solid. For this analysis, 1000 permutations were utilized to calculate the *p* values, and the permutation type was set to phenotype. Significant gene sets were defined as follows: false discovery rate (FDR) < 0.2; family wise-error rate (FWER) < 0.4; *p* < 0.05.

We analyzed tumor-infiltrating lymphocytes (TILs) using deep learning-based lymphocyte classification with convolutional neural networks (CNNs) in whole-slide image analysis and identified immune subtypes using CIBERSORT and Kallisto software and algorithms^21,27–29^.

Pathway-based network analysis was based on the identified genes linked to each histological type using Cytoscape (version 3.7.2) network visualization software. To visualize the biological relevance of the histological subtypes and their relevant elements on the basis of GSEA, we performed functional enrichment analyses using ClueGO, an application within Cytoscape software^23,24^.

### Data extraction from the GDSC and COSMIC databases

Drug screening was performed using datasets from the GDSC and COSMIC databases, which are large-scale cancer cell line and drug response databases of 988 cancer cell lines and 518 anticancer drugs, respectively. In 7 NSCLC cell lines, the drug response was measured by the natural log half-maximal inhibitory concentration (LN IC50). A drug was defined as a sensitive drug when the LN IC50 decreased in lung cell lines with a high expression of the identified genes associated with the histological types but increased in those with a low expression of the identified genes.

### Statistical analysis

Student’s t-test and/or Pearson’s correlation were used to examine the differences or relationships among continuous variables. In the TCGA database, disease-free survival (DFS) and disease-specific survival (DSS) were analyzed and generated using the Kaplan–Meier method and compared using the log rank test. A two-tailed *p* value < 0.05 was considered statistically significant. All data were analyzed using R packages and SPSS statistics (version 26.0, SPSS Inc., Chicago, IL, USA).

## Results

### Gene set enrichment analysis of the histological types of lung adenocarcinoma

**Figure 1:**
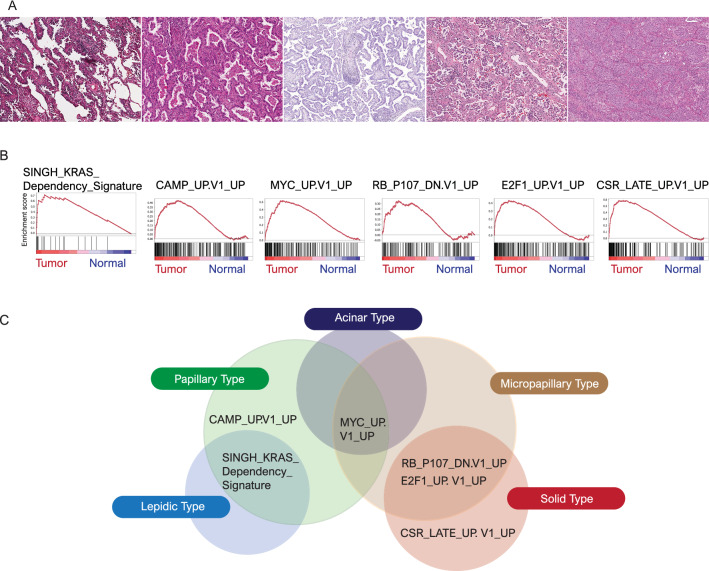
(**A**) Representative histological features showing the lepidic (left), acinar, papillary, micropapillary and solid (right) patterns. (**B**) Six gene sets, including SINGH KRAS Dependency Signature, CAMP UP.V1 UP, MYC UP.V1 UP, RB P107 DN.V1 DN, E2F1 UP.V1 UP and CSR LATE UP.V1 UP, associated with lung adenocarcinoma. (**C**) Venn diagram showing the distribution of gene sets according to the five histological types.

In TCGA, the distributions of the five predominant histological types were as follows: 38 lepidic type (7.4%), 118 acinar type (22.8%), 96 papillary type (18.6%), 54 micropapillary type (10.4%) and 211 solid type (40.8%). Of the solid type cases, 40 were associated with coagulative necrosis (Fig. 1A).

We performed GSEA to identify various gene sets associated with the lepidic, acinar, papillary, micropapillary and solid types. In the analyses of oncogenic gene sets, we found six gene sets (SINGH KRAS Dependency Signature, CAMP UP.V1 UP, MYC UP.V1 UP, RB P107 DN.V1 UP, E2F1 UP.V1 UP and CSR LATE UP.V1 UP) associated with lung adenocarcinoma (Fig. 1B). The lepidic type was linked to the SINGH KRAS Dependency Signature. The papillary type was linked to three gene sets, the SINGH KRAS Dependency Signature, CAMP UP.V1 UP and MYC UP.V1 UP. The acinar type was related to only MYC UP.V1 UP. The micropapillary type was associated with MYC UP.V1 UP, RB P107 DN.V1 UP, and E2F1 UP.V1 UP. The solid type was linked to RB P107 DN.V1 UP, E2F1 UP.V1 UP and CSR LATE UP.V1 UP. Some gene sets were shared among the histological types as follows: lepidic and papillary types: SINGH KRAS Dependency Signature; papillary, acinar, and micropapillary types: MYC UP.V1 UP; and micropapillary and solid types: E2F1 UP.V1 UP and CSR LATE UP.V1 UP (Fig. 1C) (Supplementary Table S1).

### Survival analyses according to histological type

**Figure 2:**
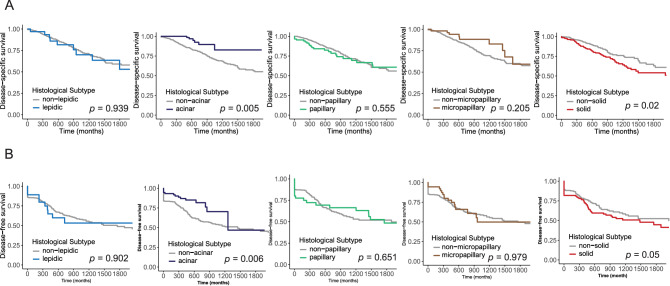
Survival analyses of the five types. (**A**) Disease-specific survival: lepidic, acinar, papillary, micropapillary and solid types (*p* = 0.939, 0.005, 0.555, 0.205 and 0.02, respectively). (**B**) Disease-free survival: lepidic, acinar, papillary, micropapillary and solid types (*p* = 0.902, 0.006, 0.651, 0.979 and 0.05, respectively).

The lepidic type was associated with unfavorable DSS and favorable DFS, but the associations were not significant (*p* = 0.939 and 0.902, respectively). The acinar type was significantly related to longer DSS and DFS than the other types (*p* = 0.005 and 0.006, respectively). There were no differences in DSS and DFS between the papillary type and the other types (*p* = 0.555 and 0.651, respectively). The micropapillary type correlated with good DSS and worse DFS, but the correlations were not significant (*p* = 0.205 and 0.979, respectively). The solid type was significantly associated with shorter DSS and DFS than the other types (*p* = 0.02 and 0.05, respectively) (Fig. 2). On the basis of these results, we focused on the solid type, which had the poorest prognosis.

### Immune cell profiles and tumor cell proliferation and antigenicity in the solid type

In the analyses of the solid type, we referred to immune cell profiles, tumor cell proliferation and tumor antigenicity based on a study by Thorsson et al. and in silico cytometry^28^.

**Figure 3:**
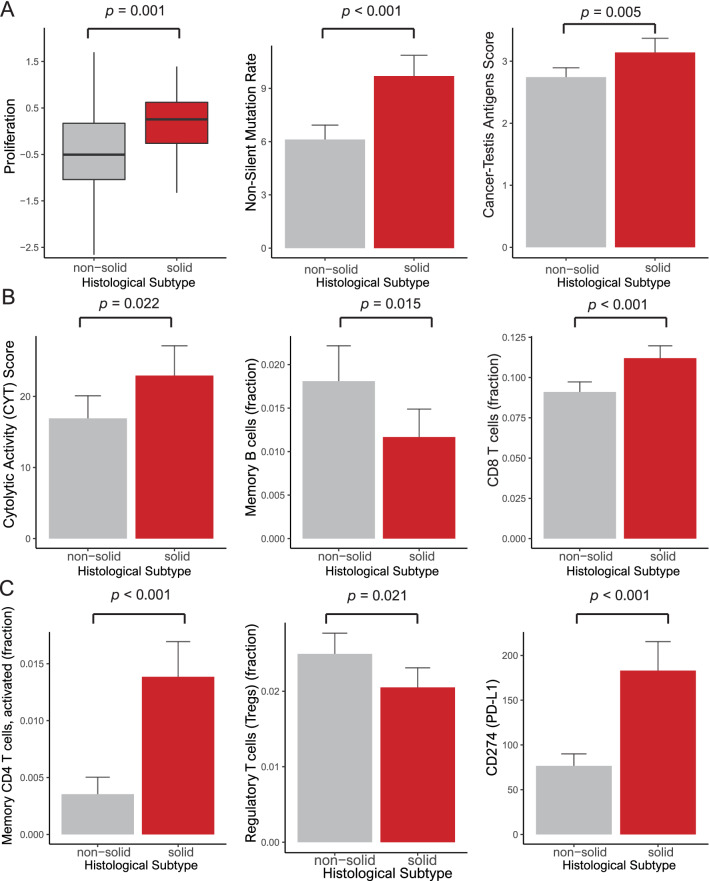
Bar plots of the solid type. (**A**) Proliferation, non-silent mutation rate and cancer-testis antigen score (*p* = 0.001, < 0.001 and 0.005, respectively). (**B**) Cytolytic activity score, memory B cells and CD8 T cells (*p* = 0.022, 0.015 and < 0.001, respectively). (**C**) Activated memory CD4 T cells, regulatory T cells and CD274 (PD-L1) (*p* < 0.001, 0.021 and < 0.001, respectively).

The solid type was associated with higher proliferation, nonsilent mutation rates known as a part of tumor mutation burden, cancer-testis antigen (CTA) scores and cytolytic activity than the other types (*p* = 0.001, < 0.001, 0.005 and 0.022, respectively). In comparing the immune cell fractions between the solid type and the other types, memory B cells and regulatory T cells were decreased in the solid type (*p* = 0.015 and 0.021, respectively), while CD8 T cells and activated memory CD4 T cells were increased in the solid type (*p* < 0.001 and 0.001, respectively). CD274 (encoding PD-L1) expression was more elevated in the solid type than in the other types (*p* < 0.001) (Fig. 3).

### Pathway-based network analysis and genetic alterations in the solid type

**Figure 4:**
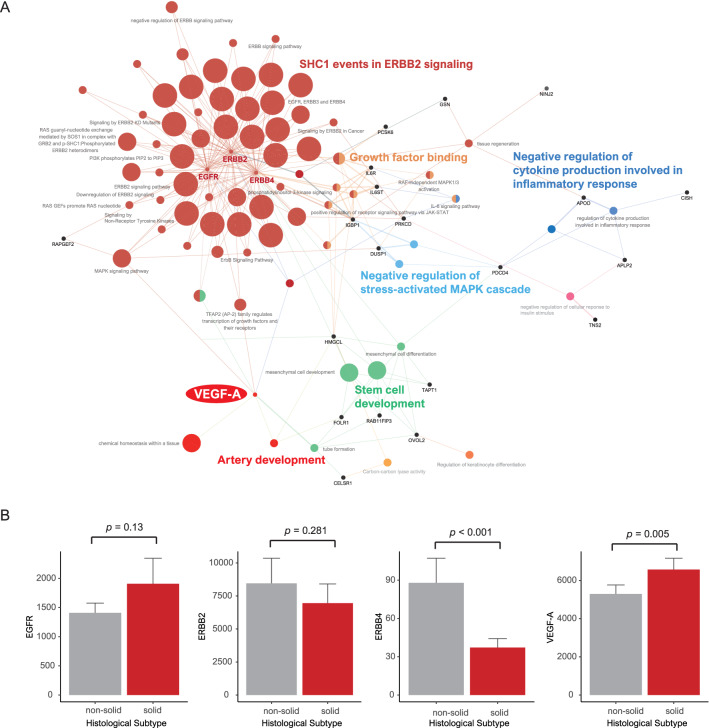
(**A**) Grouping of networks based on functionally enriched Gene Ontology (GO) terms and pathways in the solid type. The functionally grouped networks are linked to their biological function, where only the most significant term in the group is labeled. (**B**) Bar plots of EGFR, ERBB2, ERBB4 and VEGF-A (*p* = 0.13, 0.281 < 0.001 and 0.005, respectively).

We performed pathway-based network analysis using the genes and gene sets associated with the solid type. The solid type was linked to 6 functionally enriched Gene Ontology (GO) terms and pathways: (1) SHC1, which is related to the regulation of apoptosis and drug resistance events in ERBB2 signaling (also known as human epidermal growth factor receptor 2, HER-2); 2) growth factor binding; (3) negative regulation of cytokine production involved in the inflammatory response; (4) negative regulation of the stress-activated mitogen-activated protein kinase (MAPK) cascade; ((5) artery development; and (6) stem cell development (Fig. 4A). Intriguingly, the GO analysis revealed that the solid type was related to growth factor receptor tyrosine kinase and blood vessel development, reflecting cancer proliferation. We focused on epidermal growth factor receptor (EGFR), ERBB2, ERBB4-related ERBB2 signaling and vascular endothelial growth factor (VEGF)-A associated with blood vessel development in the solid type.

EGFR expression was higher in the solid type than in the other types, but it was not significant (*p* = 0.13). ERBB2 and ERBB4 expression was lower in the solid type than in the other types (*p* = 0.281 and < 0.001, respectively). VEGF-A was more elevated in the solid type than in the other types (*p* = 0.005). On the basis of these results, we focused on VEGF-A reflecting tumor blood vessel development in the solid type (Fig. 4B).

### Drug screening of lung cancer cell lines

**Figure 5:**
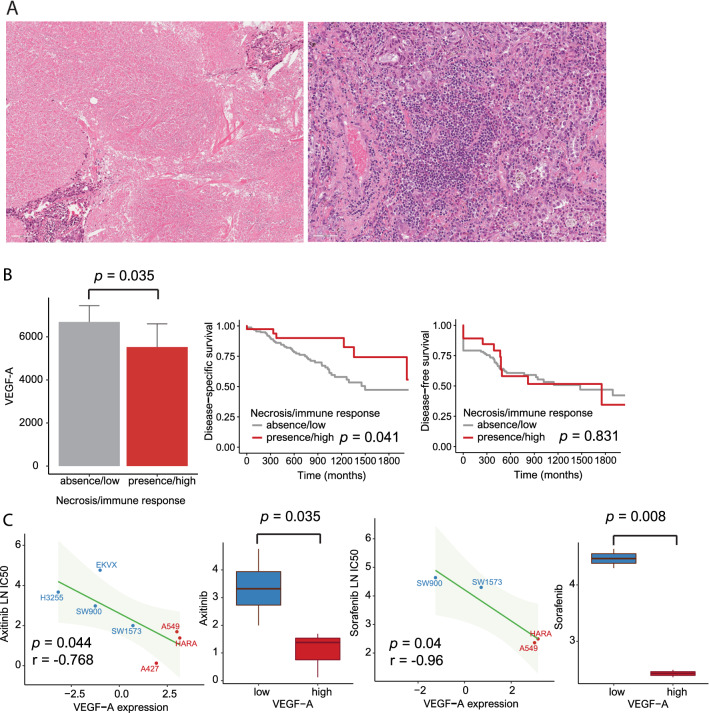
(**A**) Representative histological features: coagulative necrosis (left) and tumor-infiltrating lymphocytes (TILs) (right). (**B**) Bar plots of VEGF-A according to necrosis (coagulative necrosis)/immune response↑ (elevated TILs) (*p* = 0.035) (left) and disease-specific survival/disease-free survival according to necrosis/immune response↑ (*p* = 0.041 and 0.831, respectively). (**C**) Pearson’s correlations and boxplots showing the natural log half-maximal inhibitory concentration (LN IC50) of axitinib [r =  − 0.768, *p* = 0.044 (Pearson’s correlation) and 0.035 (Student’s t-test)] (left) and sorafenib [r =  − 0.96, *p* = 0.04 (Pearson’s correlation) and 0.008 (Student’s t-test)] (right).

In the solid type with high VEGF-A expression, we evaluated the histological features of coagulative necrosis related to ischemic changes and TILs related to the immune response. VEGF-A expression was decreased in the presence of coagulative necrosis and elevated TILs (*p* = 0.035). Elevated TILs and the presence of coagulative necrosis were associated with better DSS than reduced TILs and the absence of coagulative necrosis (*p* = 0.041). There was no significant relationship between DFS and TILs/coagulative necrosis (*p* = 0.831) (Fig. 5A,B) (Supplementary Table S2).

Axitinib and sorafenib, as VEGF inhibitors, effectively suppressed the growth of lung cancer cells with high VEGF-A expression. Sorafenib greatly inhibited the proliferation of cells with high VEGF-A expression compared to the inhibitory effect of axitinib [sorafenib: r =  − 0.96, *p* = 0.04 (Pearson’s correlation) and 0.008 (Student’s t-test); axitinib: r =  − 0.768, *p* = 0.044 (Pearson’s correlation) and 0.035 (Student’s t-test)] (Fig. 5C).

## Discussion

This study showed survival differences among the five histological types and focused on genetic/molecular alterations in patients with lung adenocarcinoma. In previous studies, the simple histological grading system, involving the predominant histological type, has been shown to correlate with prognosis^30,31^. Some studies suggested the importance of architectural grading^32,33^, nuclear grading^34,35^, or both^36^, but the most applicable grading involved the following three architectural grades: grade 1 (well differentiated), lepidic; grade 2 (moderately differentiated), acinar or papillary; and grade 3 (poorly differentiated), micropapillary and solid. We classified the three-tiered grading system into five histological types and analyzed survival rates and oncogenic gene sets accordingly. A total of six gene sets associated with the histological types were identified, and some types shared gene sets, such as the SINGH KRAS Dependency Signature, MYC UP.V1 UP, RB P107 DN.V1 DN and E2F1 UP.V1 UP. The presence of gene sets shared by multiple histological types could mean that they partially share common elements which may results in the heterogeneous composition of lung adenocarcinoma. More than 2 histological types were observed by three reviewers (KM, WJ and YO) in one predominant type, and there were some findings in which it was difficult to select the predominant type^37^. In other words, the sharing of gene sets could be thought to be similar to the aspect that a large number of lung adenocarcinomas share two or more histological features. In the survival analyses, the acinar type had better DSS and DFS than the other types, whereas the solid type had poorer DSS and DFS than the other types. Unexpectedly, there were no significant differences in DSS and DFS between the other types and the lepidic, papillary or micropapillary types. These results suggest that the histological classification of lung adenocarcinoma may have limitations in predicting clinical prognosis.

This study focused on the solid type, which was observed most frequently (40.8%) and had the worst prognosis. The solid type was associated with high tumor cell proliferation and hypermutation compared with the other types. A few solid type cases showed histological features that progressed in the form of filling the acinar lumen, which is thought to accumulate more somatic mutations for a longer period than the other types. We presumed an increase in antigenic variations arising from the accumulation of somatic mutations. In addition, the CTA score was elevated with high cytolytic activity. In the solid type, the results could be interpreted as hypermutation, and a high CTA score recruits immune cells; then, the anticancer immune response induces the direct lysis of normal and/or tumor areas surrounding the invading adenocarcinoma. Interestingly, the solid type was related to an increase in immune cells, such as CD8 T cells and activated memory CD4 T cells, whereas it was associated with a decrease in other immune cells, such as memory B cells and regulatory T cells. The antigens of solid tumors have a greater effect on increasing CD8 or CD4 T cells than different types of immune cells. Notably, CD274 (encoding PD-L1) expression was highly elevated in the solid type compared to the other types. This is considered a defense mechanism to escape anticancer immune response attacks via elevated CD8 T cells. In other words, adaptive immune resistance, known as the type I tumor microenvironment, may have the benefit of anti-PD-L1 therapy to disable the effects of PD-L1, which suppresses the antitumoral activity of CD8 T cells^38^.

The major GO terms associated with the solid type using a pathway-based network were “ERBB2 signaling” related to growth factor tyrosine kinase and “artery development” associated with angiogenesis. Artery development is not just a single processing stage of angiogenesis, but is made by various signaling pathways and/or elements in numerous steps.

In comparing the solid type and the other types, EGFR was elevated, whereas ERBB2 and ERBB4 were reduced in the solid type. This finding suggests that EGFR could play an important role in the growth of the solid type. A previous study described a tumor suppressor function for ERBB4 known as HER4 in triple-negative breast carcinoma^39^. However, another study demonstrated that ERBB4 could promote the progression of colorectal adenocarcinoma through epithelial-mesenchymal transition^40^. Although ERBB4 as a prognostic marker has been debated, the morphology of the solid type is similar to invasive ductal carcinoma of triple-negative breast carcinoma compared to colorectal adenocarcinoma. Thus, we hypothesized that ERBB4 functions as a tumor suppressor in the solid type of lung adenocarcinoma.

Notably, VEGF-A expression was highly increased in the solid type compared to the other types. VEGF-A is an important biomarker that stimulates angiogenesis and could provide an essential environment for cancer cell growth^41^. Moreover, the effect of VEGF-A could inhibit different immune cells with T cells as well as thymic atrophy^42–44^. On the basis of these functions, our study focused on high VEGF-A expression in the solid type. We investigated coagulative necrosis reflecting ischemic changes and TILs correlating to the function of VEGF in the solid type. The presence of coagulative necrosis and elevated TILs was associated with low VEGF-A expression compared to the absence of coagulative necrosis and reduced TILs. In the survival analysis, the presence of coagulative necrosis and increased TILs was related to longer DSS than the absence of coagulative necrosis and decreased TILs in the solid type. Subsequently, we evaluated the extent of the inhibition of VEGF functions by axitinib and sorafenib as VEGF inhibitors and its effect on the survival rate of lung cancer cells with high VEGF-A expression^45^. The cancer cells were significantly reduced by the VEGF inhibitors. Previous studies demonstrated that VEGF can act by selective autocrine effects to stimulate tumor cell proliferation, survival, adhesion and chemotaxis in various malignancies such as breast, stomach and skin^46–48^. These results support that VEGF-A plays a pivotal role in prognosis in the solid type.

This study has several limitations that should be acknowledged. First, because this is a cross-sectional study and in silico analyses from the TCGA and GDSC databases did not show sustained relationships over time, it is difficult to come to a definitive conclusion. Second, experimental data allowing for novel biological insights of the solid type associated with high VEGF-A were not shown in our study. We should consider that tissue microarray (for immunohistochemistry) and blood vessel-specific marker (for vascular density) was needed for further evaluation. Further in vitro and/or in vivo studies may be necessary to clarify the molecular mechanisms in the solid type of lung adenocarcinoma. Third, pharmacokinetics in lung cells may be highly heterogeneous in solid-type patients with various pharmacodynamics affected by disease status, microenvironments, and immunities.

This study demonstrated that the solid type was associated with increased tumor cell proliferation, hypermutations, and CTA scores, which produced an unfavorable prognosis in patients with lung adenocarcinoma. This finding implies that the increased antigenicity of the solid type through hypermutation could enhance recruitment of immune cells such as CD8 T cells and activated memory CD4 T cells. The increased CD8+ T cells and high CD274 expression in patients with solid-type disease could be the result of adaptive immune resistance, which is an indicator of anti-PD-L1 therapies. The pathway-based network analysis revealed a significant relationship between the solid type and high VEGF-A expression. Solid type cases with elevated VEGF-A levels were associated with poorer prognosis than those with reduced VEGF-A expression. The VEGF-A inhibitor showed a response against lung cancer cells with high VEGF-A expression and could be a treatment option for solid-type cases with increased VEGF-A levels.

Our workflow results will contribute to designing future experimental studies and drug development in patients with the solid type of lung adenocarcinoma.

## Supplementary Information

Supplementary Information.

## Data Availability

The authors declare that all data supporting the findings of this study are available within the article.
