# Supplementary material for: Increased VEGF-A in solid type of lung adenocarcinoma reduces the patients’ survival
Source: Sci Rep. 2021 Jan 14;11:1321. doi: 10.1038/s41598-020-79907-6 (PMC7809025; doi:10.1038/s41598-020-79907-6)
Supplement: Supplementary file 1 — Supplementary Information. [file 41598_2020_79907_MOESM1_ESM.docx]

**Increased VEGF-A in solid type of lung adenocarcinoma reduces the patients' survival**

Woon Yong Jung^1^, Kyueng-Whan Min^1*^, Young Ha Oh^1^

^1^ Department of Pathology, Hanyang University Guri Hospital, Hanyang University College of Medicine, Guri, Gyeonggi-do, Republic of Korea

**Running title:** Solid type of lung adenocarcinoma

***Correspondence to:**

Kyueng-Whan Min, M.D., Ph.D.

Department of Pathology, Hanyang University Guri Hospital, Hanyang University College of Medicine,

Kyoungchun-ro 153, Guri-si, Gyeonggi-do 11923, Republic of Korea

Tel: +82-31-560-2496; Fax: +82-31-560-2339

E-mail: [kyueng@gmail.com](mailto:kyueng@gmail.com)

**Supplementary table 1.** Gene sets related to lung adenocarcinoma

| **NAME** | **SIZE** | **ES** | **NES** | **NOM** | **FDR** | **FWER** |
| --- | --- | --- | --- | --- | --- | --- |
|  |  |  |  | (p-value) | (q-value) | (p-value) |
| SINGH_KRAS_DEPENDENCY_SIGNATURE | 20 | 0.71 | 1.69 | 0.003 | 0.274 | 0.242 |
| CAMP_UP.V1_UP | 198 | 0.42 | 1.71 | 0.002 | 0.056 | 0.252 |
| MYC_UP.V1_UP | 177 | 0.51 | 1.8 | 0.004 | 0.035 | 0.132 |
| RB_P107_DN.V1_UP | 140 | 0.54 | 1.74 | 0.012 | 0.045 | 0.185 |
| E2F1_UP.V1_UP | 189 | 0.43 | 1.69 | 0.006 | 0.054 | 0.267 |
| CSR_LATE_UP.V1_UP | 165 | 0.52 | 1.67 | 0.012 | 0.056 | 0.314 |
| ES, Enrichment Score; NES, Normalized Enrichment Score; FDR, False Discovery Rate; NOM, Nominal p Value; FDR, False discovery rate; FWER, Family wise-error rate | | | | | | |

**Supplementary table 2.** Data of necrosis and immune response in 211 patients with solid type

| **ID** | **Type** | **Necrosis** | **Immune response** | **Necrosis/immune response** |
| --- | --- | --- | --- | --- |
| TCGA-05-4244 | solid | absence | low | absence/low |
| TCGA-05-4250 | solid | absence | low | absence/low |
| TCGA-05-4389 | solid | presence | high | presence/high |
| TCGA-05-4395 | solid | presence | low | absence/low |
| TCGA-05-4397 | solid | absence | low | absence/low |
| TCGA-05-4415 | solid | presence | low | absence/low |
| TCGA-05-4417 | solid | presence | low | absence/low |
| TCGA-05-4418 | solid | presence | high | presence/high |
| TCGA-05-4426 | solid | presence | low | absence/low |
| TCGA-05-4427 | solid | absence | low | absence/low |
| TCGA-05-4432 | solid | presence | high | presence/high |
| TCGA-05-4434 | solid | presence | low | absence/low |
| TCGA-05-5420 | solid | presence | low | absence/low |
| TCGA-05-5428 | solid | absence | low | absence/low |
| TCGA-35-4122 | solid | absence | low | absence/low |
| TCGA-35-4123 | solid | presence | high | presence/high |
| TCGA-35-5375 | solid | presence | low | absence/low |
| TCGA-38-4625 | solid | absence | low | absence/low |
| TCGA-38-4626 | solid | absence | low | absence/low |
| TCGA-38-4627 | solid | absence | low | absence/low |
| TCGA-38-4629 | solid | absence | low | absence/low |
| TCGA-38-4630 | solid | presence | low | absence/low |
| TCGA-38-4631 | solid | presence | low | absence/low |
| TCGA-38-4632 | solid | presence | high | presence/high |
| TCGA-44-2656 | solid | absence | low | absence/low |
| TCGA-44-2659 | solid | absence | low | absence/low |
| TCGA-44-2662 | solid | absence | low | absence/low |
| TCGA-44-2668 | solid | absence | low | absence/low |
| TCGA-44-3396 | solid | absence | low | absence/low |
| TCGA-44-3398 | solid | absence | low | absence/low |
| TCGA-44-3918 | solid | absence | low | absence/low |
| TCGA-44-3919 | solid | absence | low | absence/low |
| TCGA-44-5643 | solid | absence | low | absence/low |
| TCGA-44-5644 | solid | absence | low | absence/low |
| TCGA-44-6145 | solid | presence | low | absence/low |
| TCGA-44-6777 | solid | presence | low | absence/low |
| TCGA-44-6778 | solid | absence | low | absence/low |
| TCGA-44-7660 | solid | presence | high | presence/high |
| TCGA-44-7661 | solid | absence | low | absence/low |
| TCGA-44-7662 | solid | absence | low | absence/low |
| TCGA-44-7667 | solid | presence | low | absence/low |
| TCGA-44-7669 | solid | presence | low | absence/low |
| TCGA-44-7672 | solid | absence | low | absence/low |
| TCGA-44-8119 | solid | absence | low | absence/low |
| TCGA-44-A479 | solid | absence | low | absence/low |
| TCGA-44-A47A | solid | presence | high | presence/high |
| TCGA-44-A47B | solid | absence | low | absence/low |
| TCGA-44-A47G | solid | absence | low | absence/low |
| TCGA-44-A4SS | solid | presence | high | presence/high |
| TCGA-49-6743 | solid | presence | high | presence/high |
| TCGA-49-6761 | solid | absence | low | absence/low |
| TCGA-49-6767 | solid | absence | low | absence/low |
| TCGA-49-AAR3 | solid | absence | low | absence/low |
| TCGA-49-AAR9 | solid | presence | low | absence/low |
| TCGA-49-AARE | solid | presence | high | presence/high |
| TCGA-49-AARR | solid | absence | low | absence/low |
| TCGA-4B-A93V | solid | absence | low | absence/low |
| TCGA-50-5044 | solid | absence | low | absence/low |
| TCGA-50-5045 | solid | absence | low | absence/low |
| TCGA-50-5051 | solid | absence | low | absence/low |
| TCGA-50-5066 | solid | presence | high | presence/high |
| TCGA-50-5066 | solid | presence | high | presence/high |
| TCGA-50-5072 | solid | presence | low | absence/low |
| TCGA-50-5930 | solid | presence | low | absence/low |
| TCGA-50-5931 | solid | presence | low | absence/low |
| TCGA-50-5933 | solid | presence | high | presence/high |
| TCGA-50-5936 | solid | absence | low | absence/low |
| TCGA-50-5939 | solid | absence | low | absence/low |
| TCGA-50-5941 | solid | absence | low | absence/low |
| TCGA-50-6590 | solid | presence | high | presence/high |
| TCGA-50-6591 | solid | absence | low | absence/low |
| TCGA-50-6592 | solid | presence | low | absence/low |
| TCGA-50-6593 | solid | absence | low | absence/low |
| TCGA-50-6594 | solid | absence | low | absence/low |
| TCGA-50-7109 | solid | absence | low | absence/low |
| TCGA-53-7624 | solid | presence | low | absence/low |
| TCGA-53-A4EZ | solid | presence | low | absence/low |
| TCGA-55-1594 | solid | presence | low | absence/low |
| TCGA-55-1596 | solid | presence | low | absence/low |
| TCGA-55-5899 | solid | presence | low | absence/low |
| TCGA-55-6968 | solid | presence | low | absence/low |
| TCGA-55-6969 | solid | absence | low | absence/low |
| TCGA-55-6971 | solid | absence | low | absence/low |
| TCGA-55-6975 | solid | presence | low | absence/low |
| TCGA-55-6978 | solid | absence | low | absence/low |
| TCGA-55-6979 | solid | absence | low | absence/low |
| TCGA-55-6981 | solid | absence | low | absence/low |
| TCGA-55-6982 | solid | absence | low | absence/low |
| TCGA-55-6987 | solid | presence | low | absence/low |
| TCGA-55-7227 | solid | absence | low | absence/low |
| TCGA-55-7570 | solid | presence | high | presence/high |
| TCGA-55-7576 | solid | absence | low | absence/low |
| TCGA-55-7724 | solid | presence | low | absence/low |
| TCGA-55-7726 | solid | absence | low | absence/low |
| TCGA-55-7903 | solid | presence | low | absence/low |
| TCGA-55-7910 | solid | presence | low | absence/low |
| TCGA-55-7911 | solid | absence | low | absence/low |
| TCGA-55-7994 | solid | absence | low | absence/low |
| TCGA-55-7995 | solid | presence | high | presence/high |
| TCGA-55-8085 | solid | absence | low | absence/low |
| TCGA-55-8089 | solid | absence | low | absence/low |
| TCGA-55-8090 | solid | absence | low | absence/low |
| TCGA-55-8091 | solid | presence | high | presence/high |
| TCGA-55-8204 | solid | absence | low | absence/low |
| TCGA-55-8205 | solid | absence | low | absence/low |
| TCGA-55-8299 | solid | absence | low | absence/low |
| TCGA-55-8506 | solid | absence | low | absence/low |
| TCGA-55-8510 | solid | absence | low | absence/low |
| TCGA-55-8511 | solid | absence | low | absence/low |
| TCGA-55-8614 | solid | presence | low | absence/low |
| TCGA-55-8615 | solid | presence | low | absence/low |
| TCGA-55-8620 | solid | presence | high | presence/high |
| TCGA-55-8621 | solid | absence | low | absence/low |
| TCGA-55-A48Z | solid | presence | low | absence/low |
| TCGA-55-A490 | solid | absence | low | absence/low |
| TCGA-55-A493 | solid | absence | low | absence/low |
| TCGA-55-A57B | solid | absence | low | absence/low |
| TCGA-62-8394 | solid | absence | low | absence/low |
| TCGA-62-8399 | solid | presence | low | absence/low |
| TCGA-62-8402 | solid | absence | low | absence/low |
| TCGA-62-A46O | solid | presence | low | absence/low |
| TCGA-62-A471 | solid | absence | low | absence/low |
| TCGA-64-1676 | solid | absence | low | absence/low |
| TCGA-64-1678 | solid | presence | high | presence/high |
| TCGA-64-5774 | solid | presence | high | presence/high |
| TCGA-64-5775 | solid | absence | low | absence/low |
| TCGA-64-5779 | solid | presence | high | presence/high |
| TCGA-64-5781 | solid | absence | low | absence/low |
| TCGA-64-5815 | solid | presence | high | presence/high |
| TCGA-67-3771 | solid | presence | low | absence/low |
| TCGA-67-6216 | solid | presence | high | presence/high |
| TCGA-69-7973 | solid | absence | low | absence/low |
| TCGA-69-7974 | solid | absence | low | absence/low |
| TCGA-69-7978 | solid | presence | high | presence/high |
| TCGA-69-7979 | solid | presence | low | absence/low |
| TCGA-69-8255 | solid | presence | high | presence/high |
| TCGA-69-A59K | solid | absence | low | absence/low |
| TCGA-73-4666 | solid | presence | low | absence/low |
| TCGA-73-4670 | solid | absence | low | absence/low |
| TCGA-73-A9RS | solid | absence | low | absence/low |
| TCGA-75-5122 | solid | presence | high | presence/high |
| TCGA-75-5125 | solid | presence | high | presence/high |
| TCGA-75-5126 | solid | presence | low | absence/low |
| TCGA-75-6205 | solid | absence | low | absence/low |
| TCGA-75-6207 | solid | presence | low | absence/low |
| TCGA-75-6211 | solid | presence | low | absence/low |
| TCGA-75-6214 | solid | presence | low | absence/low |
| TCGA-75-7025 | solid | absence | low | absence/low |
| TCGA-75-7027 | solid | presence | low | absence/low |
| TCGA-78-7146 | solid | presence | low | absence/low |
| TCGA-78-7150 | solid | presence | low | absence/low |
| TCGA-78-7155 | solid | absence | low | absence/low |
| TCGA-78-7161 | solid | presence | low | absence/low |
| TCGA-78-7220 | solid | presence | low | absence/low |
| TCGA-78-7535 | solid | absence | low | absence/low |
| TCGA-78-7542 | solid | presence | low | absence/low |
| TCGA-78-8640 | solid | absence | low | absence/low |
| TCGA-78-8648 | solid | presence | high | presence/high |
| TCGA-80-5611 | solid | presence | high | presence/high |
| TCGA-86-7711 | solid | absence | low | absence/low |
| TCGA-86-7953 | solid | absence | low | absence/low |
| TCGA-86-7954 | solid | absence | low | absence/low |
| TCGA-86-7955 | solid | presence | high | presence/high |
| TCGA-86-8054 | solid | presence | low | absence/low |
| TCGA-86-8358 | solid | presence | low | absence/low |
| TCGA-86-8585 | solid | presence | high | presence/high |
| TCGA-86-8672 | solid | presence | low | absence/low |
| TCGA-86-A4D0 | solid | presence | high | presence/high |
| TCGA-86-A4JF | solid | absence | low | absence/low |
| TCGA-91-6830 | solid | presence | low | absence/low |
| TCGA-91-6831 | solid | presence | high | presence/high |
| TCGA-91-6835 | solid | presence | high | presence/high |
| TCGA-91-6836 | solid | absence | low | absence/low |
| TCGA-91-6848 | solid | presence | low | absence/low |
| TCGA-91-8499 | solid | presence | low | absence/low |
| TCGA-91-A4BC | solid | absence | low | absence/low |
| TCGA-93-7347 | solid | absence | low | absence/low |
| TCGA-93-A4JO | solid | absence | low | absence/low |
| TCGA-95-7043 | solid | absence | low | absence/low |
| TCGA-95-7562 | solid | presence | high | presence/high |
| TCGA-95-7944 | solid | presence | high | presence/high |
| TCGA-95-8494 | solid | presence | low | absence/low |
| TCGA-95-A4VN | solid | absence | low | absence/low |
| TCGA-95-A4VP | solid | presence | high | presence/high |
| TCGA-97-8175 | solid | absence | low | absence/low |
| TCGA-99-8025 | solid | absence | low | absence/low |
| TCGA-99-8028 | solid | absence | low | absence/low |
| TCGA-99-AA5R | solid | absence | low | absence/low |
| TCGA-L4-A4E6 | solid | absence | low | absence/low |
| TCGA-L9-A444 | solid | absence | low | absence/low |
| TCGA-L9-A5IP | solid | presence | low | absence/low |
| TCGA-L9-A743 | solid | absence | low | absence/low |
| TCGA-L9-A8F4 | solid | presence | high | presence/high |
| TCGA-MP-A4SY | solid | presence | high | presence/high |
| TCGA-MP-A4T4 | solid | absence | low | absence/low |
| TCGA-MP-A4T9 | solid | absence | low | absence/low |
| TCGA-MP-A4TF | solid | presence | high | presence/high |
| TCGA-MP-A4TI | solid | absence | low | absence/low |
| TCGA-MP-A4TK | solid | absence | low | absence/low |
| TCGA-NJ-A4YQ | solid | absence | low | absence/low |
| TCGA-NJ-A55R | solid | presence | low | absence/low |
| TCGA-44-2665 | solid | absence | low | absence/low |
| TCGA-49-AARQ | solid | absence | low | absence/low |
| TCGA-55-1595 | solid | absence | low | absence/low |
| TCGA-55-7815 | solid | absence | low | absence/low |
| TCGA-55-A4DF | solid | absence | low | absence/low |
| TCGA-62-A470 | solid | absence | low | absence/low |
| TCGA-73-4659 | solid | absence | low | absence/low |
| TCGA-86-6851 | solid | absence | low | absence/low |
| TCGA-86-7701 | solid | absence | low | absence/low |
| TCGA-95-A4VP | solid | presence | high | presence/high |
| TCGA-97-7552 | solid | absence | low | absence/low |
